# Biomarker immunoprofile in salivary duct carcinomas: clinicopathological and prognostic implications with evaluation of the revised classification

**DOI:** 10.18632/oncotarget.19812

**Published:** 2017-08-02

**Authors:** Soichiro Takase, Satoshi Kano, Yuichiro Tada, Daisuke Kawakita, Tomotaka Shimura, Hideaki Hirai, Kiyoaki Tsukahara, Akira Shimizu, Yorihisa Imanishi, Hiroyuki Ozawa, Kenji Okami, Yuichiro Sato, Yukiko Sato, Chihiro Fushimi, Takuro Okada, Hiroki Sato, Kuninori Otsuka, Yoshihiro Watanabe, Akihiro Sakai, Koji Ebisumoto, Takafumi Togashi, Yushi Ueki, Hisayuki Ota, Toyoyuki Hanazawa, Hideaki Chazono, Robert Yoshiyuki Osamura, Toshitaka Nagao

**Affiliations:** ^1^ Department of Otolaryngology Head and Neck Surgery, Tokyo Medical University, Tokyo, Japan; ^2^ Department of Anatomic Pathology, Tokyo Medical University, Tokyo, Japan; ^3^ Department of Otolaryngology-Head and Neck Surgery, Hokkaido University Graduate School of Medicine, Sapporo, Japan; ^4^ Department of Head and Neck Oncology and Surgery, International University of Health and Welfare Mita Hospital, Tokyo, Japan; ^5^ Department of Otolaryngology Head and Neck Surgery, Nagoya City University Graduate School of Medical Sciences, Nagoya, Japan; ^6^ Department of Otorhinolaryngology, Showa University School of Medicine, Tokyo, Japan; ^7^ Department of Otorhinolaryngology-Head and Neck Surgery, Keio University School of Medicine, Tokyo, Japan; ^8^ Department of Otolaryngology Head and Neck Surgery, Tokai University School of Medicine, Isehara, Japan; ^9^ Department of Head and Neck Surgery, Niigata Cancer Center Hospital, Niigata, Japan; ^10^ Department of Pathology, Cancer Institute Hospital, Japanese Foundation for Cancer Research, Tokyo, Japan; ^11^ Department of Otolaryngology, Head and Neck Surgery, Chiba University Graduate School of Medicine, Chiba, Japan; ^12^ Diagnostic Pathology Center, International University of Health and Welfare Mita Hospital, Tokyo, Japan

**Keywords:** salivary duct carcinoma, androgen receptor, p53, CK5/6, HER2

## Abstract

Salivary duct carcinoma (SDC) is an uncommon, aggressive malignant neoplasm histologically resembling high-grade mammary ductal carcinoma. SDC can arise *de novo* or ex pleomorphic adenoma. To clarify the correlation of biomarker immunoprofile with clinicopathological findings and clinical outcome of SDC, we conducted immunohistochemistry for EGFR, HER2, HER3, AR, CK5/6, p53, and Ki-67, along with HER2 fluorescence *in situ* hybridization in 151 SDCs. SDCs ex pleomorphic adenoma more commonly overexpressed EGFR, HER2, HER3, and Ki-67 than *de novo* SDCs (*P* = 0.015, < 0.001, 0.045, and 0.02, respectively). In multivariate analysis, AR− and CK5/6+ were associated with shorter progression-free survival (*P* = 0.027 and 0.004, respectively). Moreover, patients with p53-extreme negative/positive demonstrated poorer overall survival (*P* = 0.007). On assessing the revised classification by the combination of biomarker expression, the percentages of each subtype were as follows: ‘apocrine A’ (AR+/HER2−/Ki-67-low) (24%), ‘apocrine B’ (AR+/HER2−/Ki-67-high) (18%), ‘apocrine HER2’ (AR+/HER2+) (35%), ‘HER2-enriched’ (AR−/HER2+) (12%), and ‘double negative’ (AR−/HER2−) (11%). ‘Double negative’ was further subclassified into ‘basal-like’ (EGFR and/or CK5/6+) (7%) and ‘unclassified’ (3%). Consequently, patients with ‘apocrine A’ showed a better progression-free survival than those with any other subtypes. Our revised immunoprofiling classification was valuable for predicting the survival and might be useful in personalized therapy for patients with SDC.

## INTRODUCTION

Salivary duct carcinoma (SDC) is an uncommon tumor, accounting for 10% of all salivary gland carcinomas, and histologically resembles high-grade breast ductal carcinoma [1]. It occurs not only *de novo* but also as the malignant component of carcinoma ex pleomorphic adenoma (PA) [1]. The standard treatment for SDC is surgical excision and post-operative radiotherapy; however, SDC exhibits clinically aggressive behavior with locoregional recurrence and distant metastasis [2–4]. The development of salvage therapy for these patients is required in order to improve their prognosis.

Although many researchers have investigated the precise prognostic factors of SDC, there is little established evidence due to the limited number of cases for an analysis concerning this uncommon tumor entity. With respect to the clinical factors, neck involvement, tumor size, and an older age have been reported to be unfavorable prognostic factors [2–5]. Histologically, the invasive micropapillary and sarcomatoid variants are considered more aggressive SDCs [6, 7]. Thus far, however, the association between the expression of biomarkers, such as human epidermal growth factor receptor 2 (HER2), androgen receptor (AR), epidermal growth factor receptor (EGFR), p53, and Ki-67, and the patient prognosis remains controversial in SDC [5, 8–16].

Molecular subtypes defined by gene expression patterns of breast cancer using DNA microarrays are known to be of major prognostic value [17]. Subsequently, the immunohistochemical classification based on a combination of estrogen receptor (ER), progesterone receptor (PR), HER2, EGFR, CK5/6, and Ki-67 status has been developed as a surrogate; this classification system has prognostic and therapeutic implications that correlate with molecular subtypes [18, 19]. For SDCs, two immunohistochemical classification systems corresponding to the breast cancer have been suggested, but their prognostic relevance is unclarified [14, 15, 20].

In the current study, we examined the correlations of the immunoexpression of biomarkers in a large series of SDC with clinicopathological features, including the histologic origin (*i.e*., *de novo* versus ex PA), and clinical outcome. Furthermore, we attempted to propose a revised classification of SDC based on the biomarker immunoprofile and assessed its impact on the survival.

## RESULTS

### Patient characteristics

**Figure 1:**
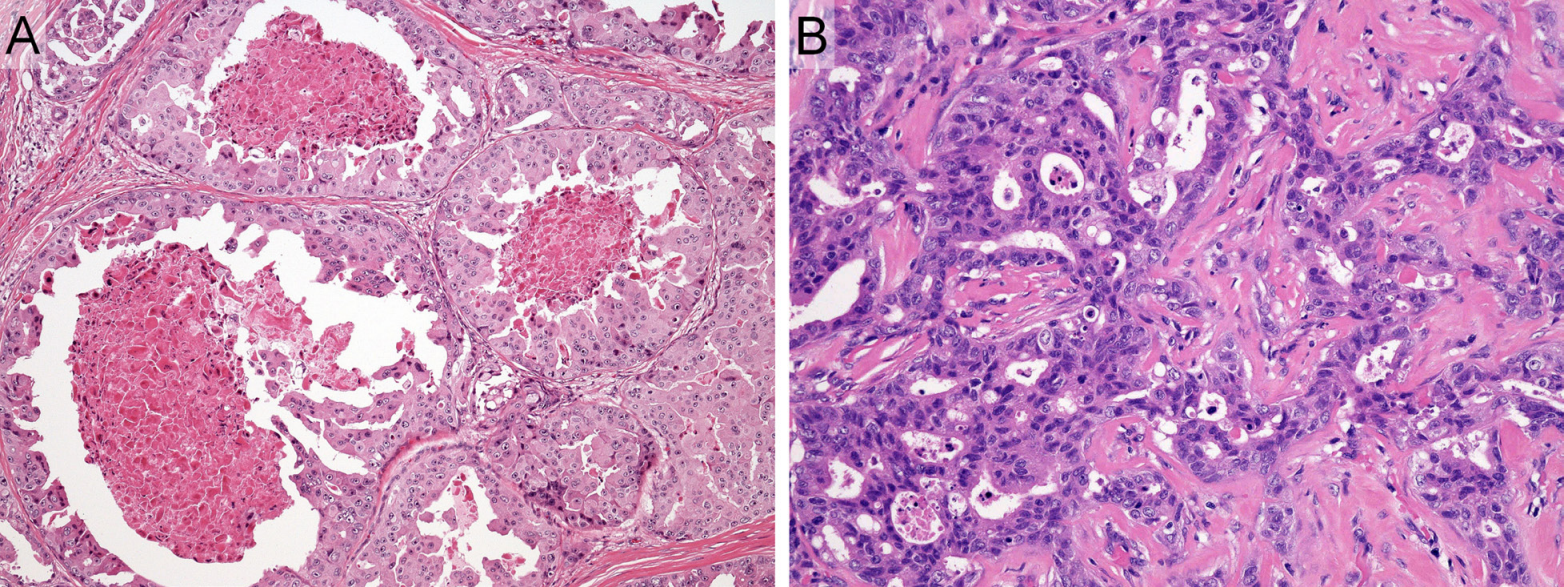
Representative histologic features of salivary duct carcinoma case (**A**) Dilated ductal structures with a papillary, “Roman-bridge,” or solid growth accompanied by comedo necrosis. (**B**) Tubular and cribriform structures with scirrhous pattern. Note that carcinoma cells display large pleomorphic nuclei and abundant eosinophilic cytoplasm.

Representative histologic features of SDC case are shown in Figure 1. The patients consisted of 127 males and 24 females, with a median age of 64 years (range, 26–87 years) (Table 1). One hundred and seventeen of 151 patients (77%) had tumors arising in the parotid gland, and 30 (20%) had tumors arising in the submandibular gland. Sixty-nine patients (46%) presented with T4 disease. Lymph node involvement was present in 80 patients (53%), and distant metastasis was observed in 9 patients (6%). Based on the histologic origin, 151 SDC cases were histologically classified as follows: 57 *de novo* cases (38%), 89 ex PA cases (59%), 5 unknown cases (3%). SDCs ex PA included 13 intracapsular SDC ex PA cases, 5 microinvasive SDC ex PA cases, and 71 widely invasive SDC ex PA cases. The median follow-up period of survivors was 3.7 years (range, 0.4–18.7 years).

**Table 1:** Patient characteristics (n = 151)

Variables	No. of patients	%
Age, years		
< 65	84	56
≥ 65	67	44
Gender		
Male	127	84
Female	24	16
T classification		
1	13	8
2	39	26
3	30	20
4	69	46
N classification		
0	71	47
1	9	6
2	71	47
M classification		
0	142	94
1	9	6
Primary tumor site		
Parotid gland	117	77
Submandibular gland	30	20
Others	4	3
First-line treatment		
Surgery	146	97
Others	5	3
Histologic origin		
*De novo*	57	38
Ex pleomorphic adenoma	89	59
Unknown	5	3

### HER2, AR, and Ki-67 status

**Figure 2:**
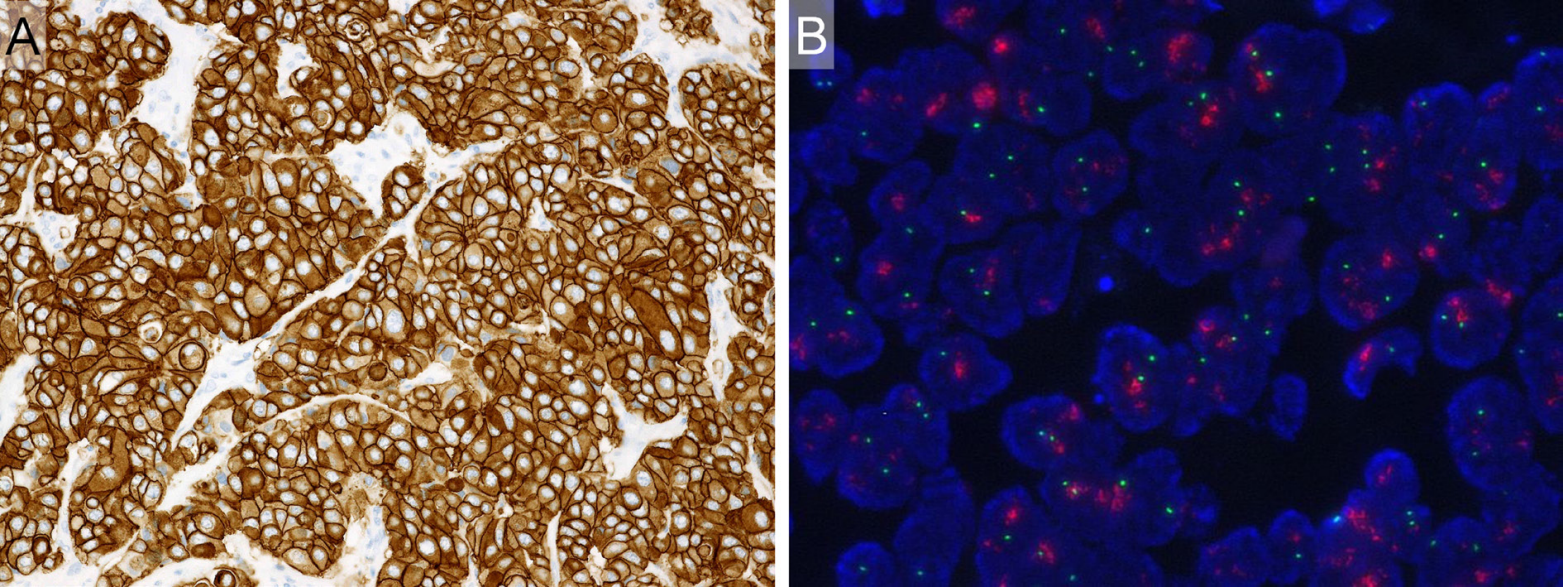
Example of HER2-positive case (**A**) Immunohistochemistry. HER2 3+. Diffuse and strong membranous staining for HER2. (**B**) Fluorescence *in situ* hybridization. Positive for *HER2* gene amplification. (HER2 genes: red signal, CEN 17: green signal).

HER2 3+ and *HER2* amplification were identified in 65 (43%) and 64 (42%) of 151 cases, respectively. In total, 70 of 151 cases (46%) were judged to be HER2 positive (Figure 2). The concordance rate of HER2 3+ and *HER2* amplification was 91% (59 of 65 cases).

**Figure 3:**
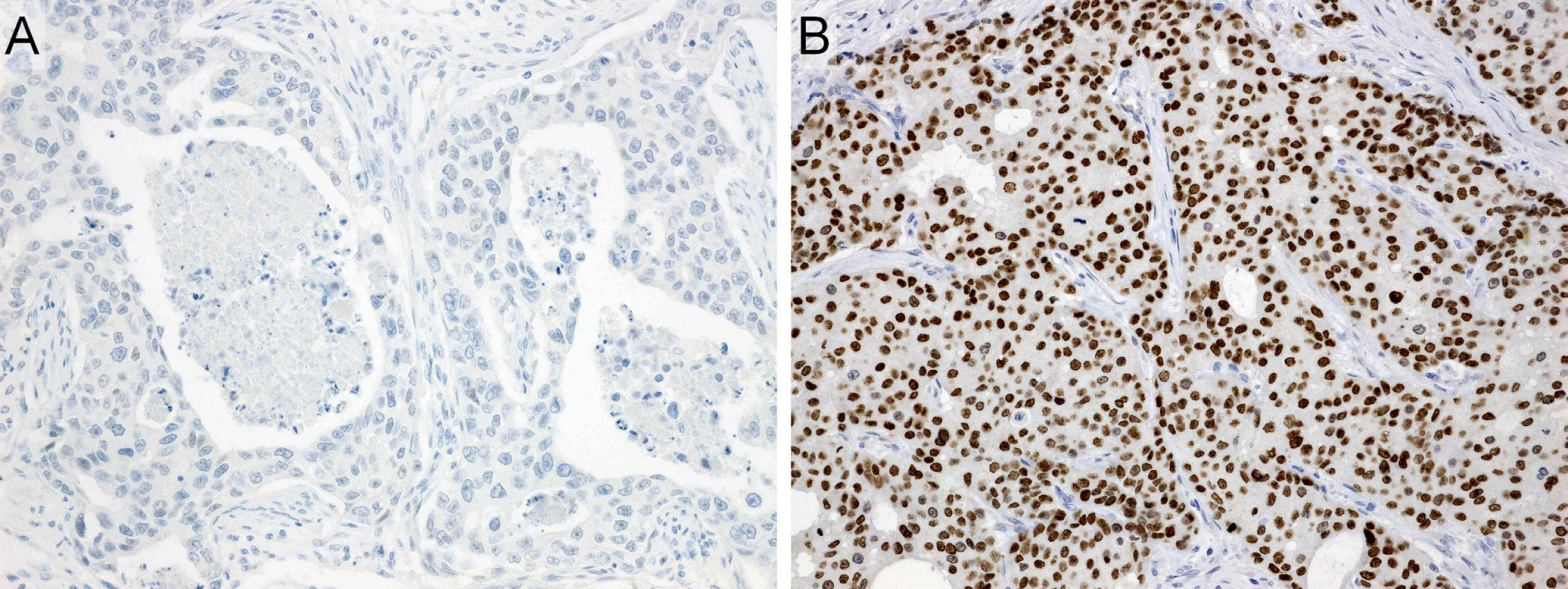
Immunohistochemistry for androgen receptor (**A**) Virtually no immunoreactivity. (**B**) Diffuse and strong nuclear immunostaining.

AR immunoreactivity was found in 144 of 150 cases (96%), and 117 of 150 cases (78%) were considered to be AR positive (Figure 3).

**Figure 4:**
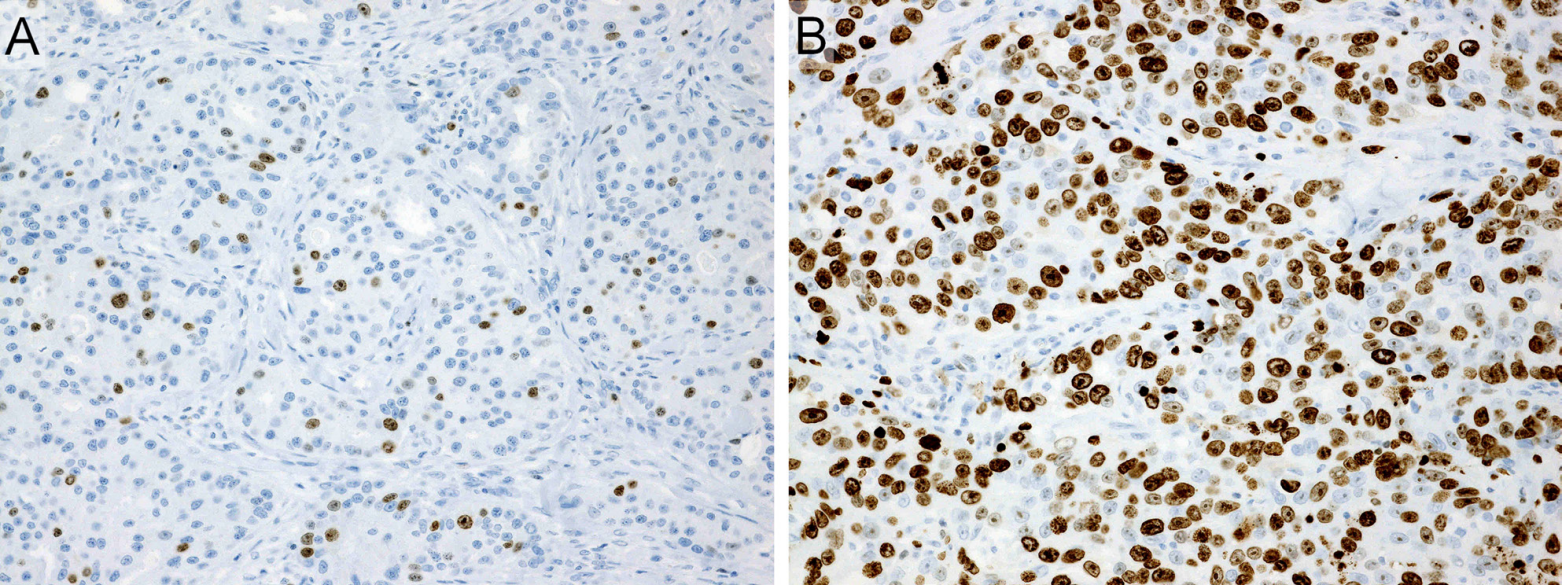
Immunohistochemistry (**A**) Ki-67-low (labeling index: 10%). (**B**) Ki-67-high (labeling index: 80%).

The mean Ki-67 labeling index (LI) was 44%, and 87 of 151 cases (58%) were categorized into high-Ki-67 LI group (Figure 4).

### Correlation of biomarker profile with clinicopathological factors

**Figure 5:**
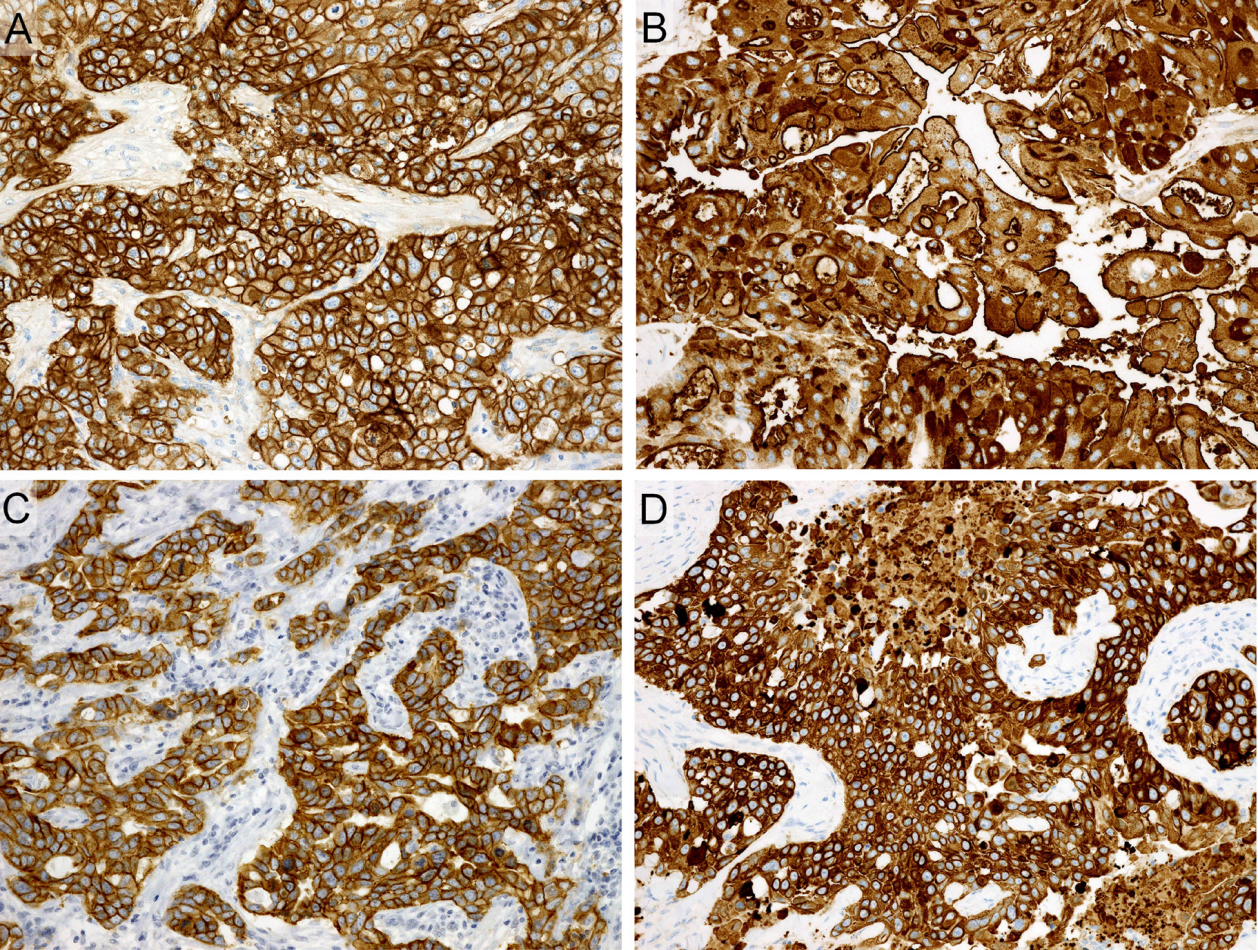
Immunohistochemistry Diffusely and strongly positive for EGFR (**A**), MUC1 (**B**), HER3 (**C**), and CK5/6 (**D**).

**Figure 6:**
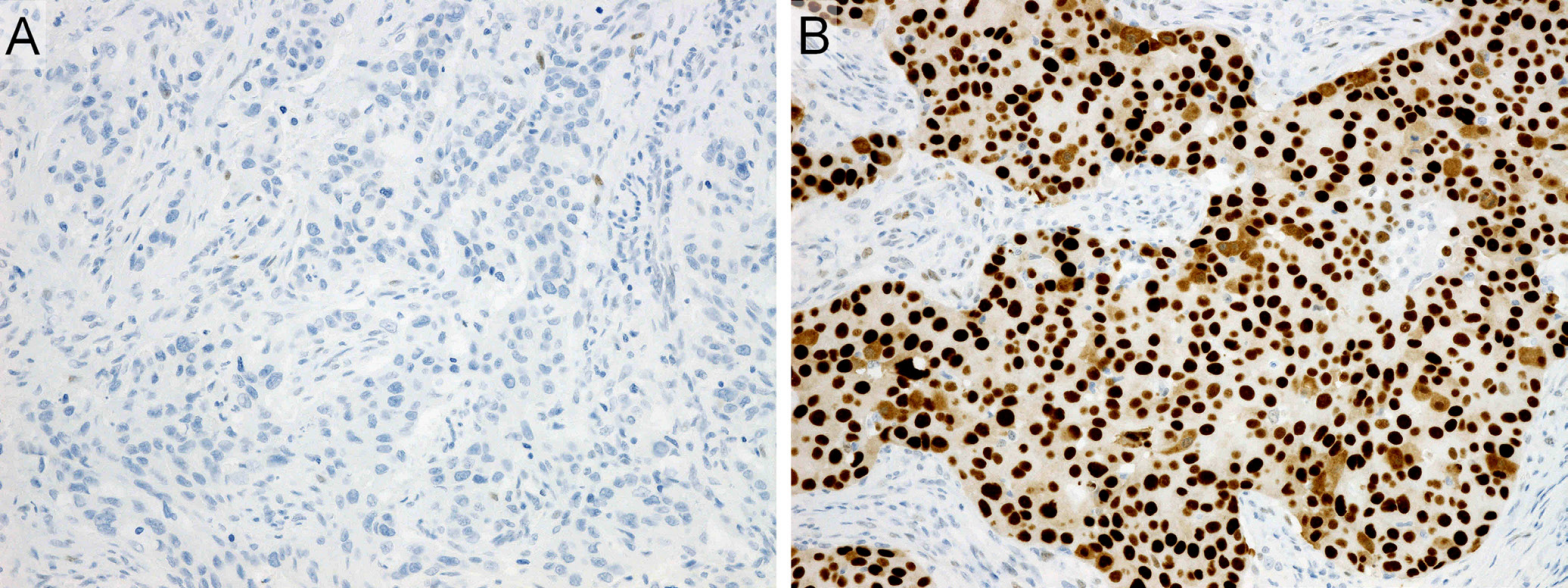
Immunohistochemistry (**A**) p53-extreme negative: carcinoma cells showing complete negativity. Note that scattered weakly positive stromal cells are observed. (**B**) p53-extreme positive: carcinoma cells showing diffuse and strong positivity.

The correlation of immunohistochemical and fluorescence *in situ* hybridization (FISH) findings of each biomarker with the clinicopathological factors are presented in Supplementary Table 1 (Figure 5). Regarding gender, AR-positive SDC more commonly developed in male patients (*P* = 0.011), while HER2-positive SDC more frequently occurred in female patients (*P* = 0.002). Regarding the TNM classification, AR-negative and HER3-negative SDCs more frequently classified as T4 (*P* = 0.021 and 0.038, respectively). Lymph node involvement presented predominantly in patients with p53-extreme negative/positive (*P* = 0.005) (Figure 6) or Ki-67-high SDC (*P* = 0.023). Distant metastasis was more frequently observed in patients with Ki-67-high SDC (*P* = 0.008). Furthermore, SDCs ex PA commonly overexpressed EGFR, HER2, HER3, and Ki-67 as compared with *de novo* SDCs (*P* = 0.015, < 0.001, 0.045, and 0.02, respectively).

### Prognostic impact of biomarkers

**Figure 7:**
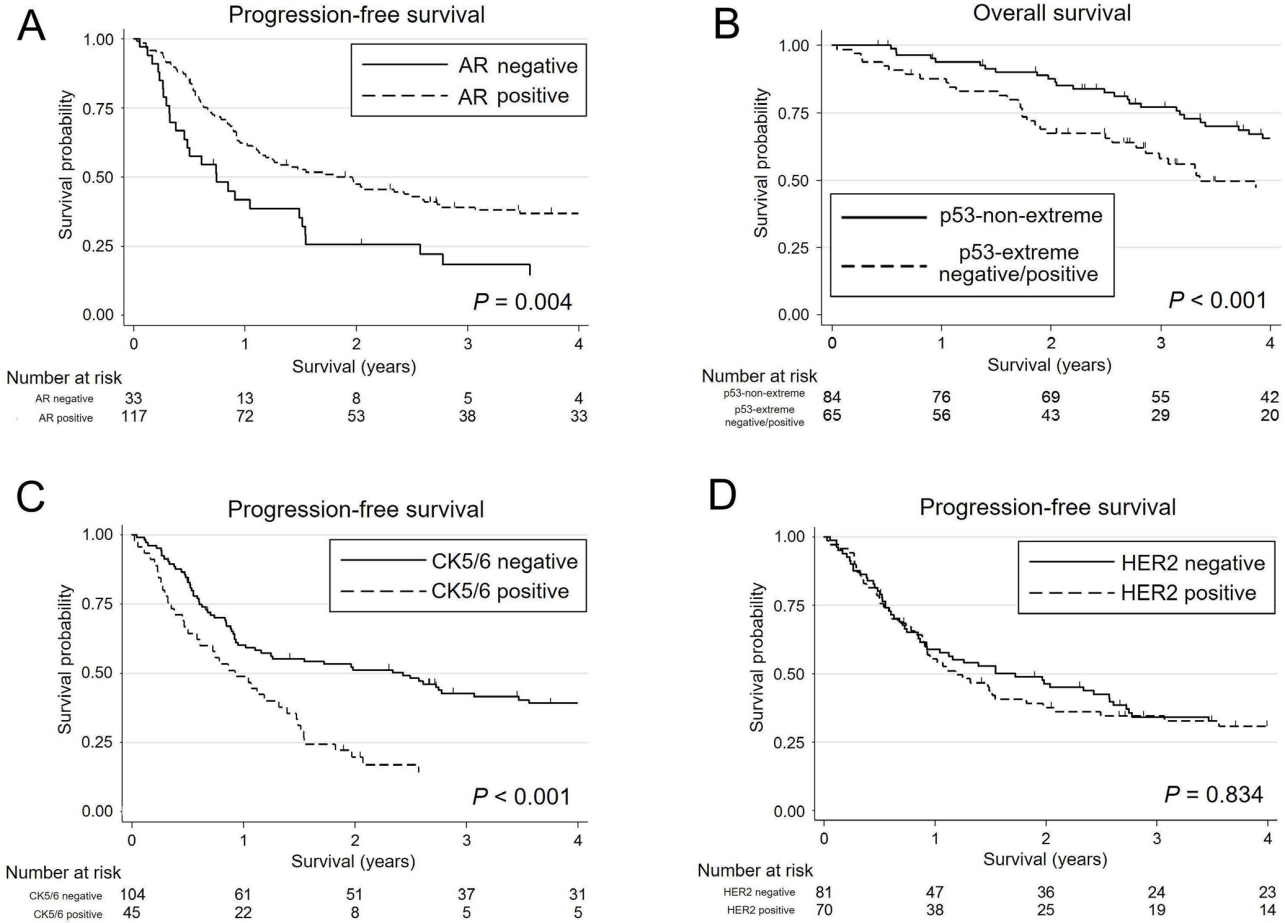
Kaplan-Meier survival curves of patients with salivary duct carcinoma (**A**) Three-year progression-free survival (PFS) rate is significantly lower for androgen receptor (AR)-negative patients (18.3%; 95% CI, 7.1–33.7) than for AR-positive patients (39.0%; 95% CI, 30.1–47.9) (*P* = 0.004). (**B**) p53-extreme negative/positive patients exhibit a significantly lower 3-year overall survival rate (58.0%; 95% CI, 44.5–69.3) than p53-non-extreme patients (77.1%; 95% CI, 66.0–84.9) (*P*<0.001). (**C**) CK5/6-positive patients show a significantly lower 3-year PFS rate (14.1%; 95% CI, 5.6–26.4) than CK5/6-negative patients (42.7%; 95% CI, 32.9–52.2) (*P* = 0.001). (**D**) There is no significant difference in 3-year PFS rate between HER2-positive (34.5%; 95% CI, 23.5–45.8) and HER2-negative patients (34.2%; 95% CI, 23.9–44.8) (*P* = 0.834).

The results of univariate and multivariate analyses of prognosis for patients with SDC are shown in Table 2. In univariate analysis, patients with AR-negative, p53-extreme negative/positive, and Ki-67-high significantly decreased both overall survival (OS) and progression-free survival (PFS). Additionally, CK5/6-positive was significantly associated with a shorter PFS. Furthermore, in the multivariate analysis, patients with p53-extreme negative/positive demonstrated a significantly worse OS. Also, AR-negative and CK5/6-positive patients were independently associated with worse PFS. Kaplan-Meier survival curves for AR, p53, and CK5/6 are shown in Figure 7A–7C.

**Table 2:** Univariate and multivariate analyses for the correlation of biomarker immunoprofile with clinical outcomes in patients with salivary duct carcinoma

Bio-markes		Overall survival						Progression-free survival					
		Univariate analysis			Multivariate analysis			Univariate analysis			Multivariate analysis		
	*n*	HR	95% CI	*P*	HR	95% CI	*P*	HR	95% CI	*P*	HR	95% CI	*P*
AR													
Neg	33	1.00	–	–	1.00	–	–	1.00	–	–	1.00	–	–
Pos	117	0.53	0.32–0.88	0.015*	0.57	0.32–1.02	0.057	0.53	0.34–0.82	0.004*	0.57	0.34–0.94	0.027*
ERβ													
Neg	5	1.00	–	–	1.00	–	–	1.00	–	–	1.00	–	–
Pos	143	1.06	0.33–3.37	0.922	0.59	0.16–2.20	0.435	1.10	0.35–3.47	0.873	0.93	0.24–3.62	0.920
EGFR													
Neg	101	1.00	–	–	1.00	–	–	1.00	–	–	1.00	–	–
Pos	50	0.90	0.55–1.49	0.693	0.67	0.39–1.17	0.161	1.03	0.69–1.54	0.881	0.86	0.55–1.36	0.525
HER2													
Neg	81	1.00	–	–	1.00	–	–	1.00	–	–	1.00	–	–
Pos	70	1.18	0.74–1.88	0.490	1.54	0.86–2.74	0.144	1.04	0.71–1.53	0.834	1.46	0.93–2.31	0.100
HER3													
Neg	48	1.00	–	–	1.00	–	–	1.00	–	–	1.00	–	–
Pos	102	0.76	0.47–1.23	0.263	0.75	0.45–1.25	0.267	0.86	0.57–1.29	0.462	0.78	0.48–1.26	0.312
MUC1													
Neg	33	1.00	–	–	1.00	–	–	1.00	–	–	1.00	–	–
Pos	115	1.19	0.67–2.11	0.547	1.49	0.82–2.73	0.191	0.84	0.54–1.33	0.461	1.10	0.68–1.79	0.698
PLAG1													
Neg	68	1.00	–	–	1.00	–	–	1.00	–	–	1.00	–	–
Pos	80	1.04	0.65–1.66	0.878	0.78	0.48–1.29	0.337	1.25	0.84–1.86	0.268	1.06	0.69–1.64	0.779
p53													
NE	84	1.00	–	–	1.00	–	–	1.00	–	–	1.00	–	–
EN/EP	65	2.50	1.55–4.02	< 0.001*	1.99	1.21–3.27	0.007*	1.84	1.25–2.71	0.002*	1.40	0.93–2.13	0.110
CK5/6													
Neg	104	1.00	–	–	1.00	–	–	1.00	–	–	1.00	–	–
Pos	45	1.50	0.91–2.47	0.111	1.38	0.80–2.38	0.253	1.99	1.33–2.99	0.001*	1.91	1.23–2.96	0.004*
Ki-67													
Low	64	1.00	–	–	1.00	–	–	1.00	–	–	1.00	–	–
High	87	1.92	1.17–3.16	0.010*	1.50	0.86–2.61	0.151	1.95	1.30–2.93	0.001*	1.49	0.93–2.39	0.099

Adjusted by age, gender, primary tumor site, TNM classification, first-line treatment, and histologic origin.

Abbreviations: HR = hazard ratio; CI = confidence interval; AR = androgen receptor; Neg = negative; Pos = positive; ER = estrogen receptor; EGFR = epidermal growth factor receptor; HER = human epidermal growth factor receptor; MUC1 = mucin-1; PLAG1 = pleomorphic adenoma gene 1; NE = not extreme; EN/EP = extreme negative/positive; CK = cytokeratin.

*Statistically significant (*P* < 0.05)

The immunoexpression of biomarkers other than those mentioned above, including ERβ, EGFR, HER2 (Figure 7D), HER3, MUC1, and PLAG1 did not have a significant impact on the survival of patients with SDC.

### Revised classification based on biomarker immunoprofiling

As shown in Table 3, the percentages for each subtype by our revised classification were ‘apocrine A’ (AR+/HER2−/Ki-67-low): 24%, ‘apocrine B’ (AR+/HER2−/Ki-67-high): 18%, ‘apocrine HER2’ (AR+/HER2+): 35%, ‘HER2-enriched’ (AR−/HER2+): 12%, and ‘double negative’ (AR−/HER2−): 11%. The ‘double negative’ subtype included 7% for ‘basal-like’ (AR−/HER2−/EGFR and/or CK5/6+) and 3% for ‘unclassified’ (others). On the other hand, the incidence of each subtype according to the classification proposed by Di Palma et al. [14] was as follows: ‘luminal AR positive’ (AR+/HER2−): 43%, ‘HER2 positive’ (AR any/HER2+): 47%, ‘basal-like’ (AR−/HER2−/EGFR and/or CK5/6+): 7%, and ‘intermediate’ (others): 3%.

**Table 3:** Univariate and multivariate analyses for clinical outcomes according to the classification based on the biomarker immunoprofiling in patients with salivary duct carcinoma

		Overall survival						Progression-free survival					
		Univariate analysis			Multivariate analysis			Univariate analysis			Multivariate analysis		
	*n* (%)	HR	95% CI	*P*	HR	95% CI	*P*	HR	95% CI	*P*	HR	95% CI	*P*
***Revised classification†***													
Apocrine A	36 (24)	1.00	–	–	1.00	–	–	1.00	–	–	1.00	–	–
Apocrine B	28 (18)	2.20	1.01–4.79	0.047*	1.75	0.78–3.92	0.178	2.49	1.33–4.68	0.004*	1.80	0.91–3.59	0.093
Apocrine HER2	53 (35)	1.87	0.93–3.78	0.080	2.19	0.97–4.95	0.058	1.92	1.09–3.39	0.025*	2.35	1.21–4.55	0.012*
HER2-enriched	17 (12)	2.60	1.14–5.91	0.023*	4.57	1.60–13.05	0.004*	2.19	1.05–4.55	0.037*	3.29	1.40–7.74	0.006*
Double negative	16 (11)	3.52	1.55–7.99	0.003*	2.36	0.94–5.90	0.067	4.80	2.42–9.49	<0.001*	3.01	1.36–6.65	0.006*
***Di Palma classification‡***													
Luminal AR positive	64 (43)	1.00	–	–	1.00	–	–	1.00	–	–	1.00	–	–
HER2 positive	70 (47)	1.45	0.87–2.42	0.156	1.83	0.97–3.45	0.061	1.32	0.87–2.01	0.194	1.82	1.11–3.00	0.019*
Basal-like	11 (7)	2.69	1.16–6.21	0.021*	1.53	0.60–3.87	0.374	3.76	1.91–7.38	<0.001*	2.63	1.22–5.68	0.014*
Intermediate	5 (3)	2.18	0.76–6.28	0.149	1.92	0.63–5.83	0.251	2.37	0.93–6.02	0.069	1.43	0.51–4.02	0.493

* Statistically significant (*P* < 0.05)

†Apocrine A, AR+/HER2−/Ki-67-low; Apocrine B, AR+/HER2−/Ki-67-high; Apocrine HER2, AR+/HER2+; HER2-enriched, AR−/HER2+; Double negative, AR−/HER2− (including basal-like [AR−/HER2−/EGFR and/or CK5/6+] and unclassified [others]).

‡Luminal AR positive, AR+/HER2−; HER2 positive, AR any/HER2+; Basal-like, AR−/HER2−/EGFR and/or CK5/6+; Intermediate, negative for all markers.

Abbreviations: HR = hazard ratio; CI = confidence interval; HER2 = human epidermal growth factor receptor 2; AR = androgen receptor; EGFR = epidermal growth factor receptor; CK = cytokeratin.

Concerning the prognostic value of our revised classification, patients with ‘apocrine A’ demonstrated a longer PFS than those of any other subtype (Table 3). Alternatively, with the Di Palma classification [14], the only remarkable finding was that patients with ‘luminal AR’ showed a better PFS than those of ‘basal-like’.

## DISCUSSION

Because SDC is an uncommon entity and frequently poses a diagnostic challenge for general pathologists, the precise immunohistochemical biomarker expression profile and its correlation with the clinicopathological and prognostic significance are not fully explored [1, 13]. Thus, thorough large-scale series investigation is necessary to establish the convincing evidence-based data for this highly aggressive tumor.

With the recent introduction of HER2-targeted therapy for patients with SDC, the determination of HER2 status is crucial in order to select patients who may benefit from this treatment [21–24]. However, the positive rate of HER2 overexpression in SDC reported previously is extremely broad, ranging from 15% to 100% [9, 11, 14, 20, 22], due seemingly to the ambiguous criteria defining the positivity. When the 2007 ASCO/CAP guideline recommending HER2 testing for breast cancer [25] is adopted for the evaluation, HER2 positivity of SDC ranges from 15% to 44% [12, 14, 15, 20, 26]. In the current study, the value of 46% was slightly higher than that in previous reports, largely because we assessed the HER2 status based on both immunohistochemistry and FISH findings in accordance with the updated 2013 ASCO/CAP guideline [27], in which the cutoff immunohistochemical level for HER2 positivity was reduced from 30% to 10%. Additionally, our results revealed that SDCs showed extremely high concordance between cases showing HER2 3+ and *HER2* amplification. Therefore, the expression of HER2 protein in SDC is highly influenced by the *HER2* amplification status.

Regarding the correlation of HER2 status with clinical features, the HER2 positivity is considered to be a predictor of a poor prognosis in breast cancer [17, 18]. In SDCs, although Skálová et al. [9] and Jaehne et al. [5] reported that HER2 overexpression was linked to a poor survival in their analysis of 11 and 34 cases, respectively, we failed to detect any relationship between the HER2 status determined by immunohistochemistry and/or FISH analysis and the clinical outcome, comparable to that found in recent studies [12, 15, 26].

SDC not only occurs *de novo* but also arises in PA [1]. However, the difference in the molecular mechanisms underlying the carcinogenesis between these two sequences is still poorly understood. Recently, Chiosea et al. have revealed that using targeted next-generation sequencing, SDCs ex PA tend to have *TP53* mutations or *ERBB2* copy number gain, whereas *de novo* SDCs frequently harbor combined *HRAS/PIK3CA* mutations but no *ERBB2* amplification [16]. We verified that SDCs ex PA commonly showed not only HER2-positive but also overexpression of EGFR and HER3 as compared with the *de novo* type. Therefore, activation of HER family members is a more crucial event in the carcinogenesis of SDC when it arises in PA than with *de novo* occurrence. A further analysis of downstream events in the HER family signaling pathway is required to clarify the detailed mechanism of carcinogenesis in SDC ex PA. We did not find statistically significant relationship between p53 expression and histologic origin of SDC.

SDCs frequently express AR [11, 13–15, 20, 28–30]. In the current study, AR immunoreactivity was identified in 96% of SDC cases, which was similar to that obtained in other series [28, 29]. Additionally, the patients with high AR-positivity rate were predominantly male. In prostate and breast cancers, AR expression has been associated with a favorable prognosis [31, 32]. However, the correlation of expression of AR with the clinical outcome of SDC has not been fully investigated [10, 15]. Williams et al. reported that patients with a combined AR−/ERβ− phenotype had a decreased survival compared with patients with combined AR+/ERβ+ or AR+/ERβ− SDC, but those with AR alone examination results were not provided [10]. Using a cutoff value of 20% nuclear staining of tumor cells, we found that AR-negative patients had a significantly worse prognosis than AR-positive patients, although the ERβ expression did not influence the survival. The role of AR in the generation or progression of SDC has been under investigated [29]; however, AR has recently become a key target of androgen deprivation treatment for this tumor [2, 11, 21, 24, 33, 34], as with prostate cancer. Further studies will be required to verify the effect and potential resistance mechanisms for this therapy.

CKs, intermediate filament proteins, reflect the epithelial cell type and state of tissue growth and differentiation in addition to the functional status of the tissue. In the breast and salivary gland, CK5/6 is regarded as a basal marker. To our knowledge, this is the first study to report that the overexpression of CK5/6 was an independent prognostic factor in patients with SDC, a finding that was equivalent to its role in a breast cancer study [18].

Whether or not the p53 expression can be a consistent independent prognostic biomarker of SDC is still under debate [5, 8, 16]. Most recently, in breast cancer, Boyle et al. attempted to classify the p53 expression patterns into three groups: extreme negative, extreme positive, and non-extreme [35]. They found that p53-extreme negative/positive expression was significantly associated with a poorer OS than p53-non-extreme expression, and that combined p53-extreme negative/positive expression better predicted the OS than either pattern alone. Furthermore, in their analysis of the *TP53* mutation status, detectable mutation types appeared to be related to the protein status, with a missense mutation corresponding to the extreme positive phenotype, and the nonsense mutation appearing to abrogate the protein expression, manifesting as the extreme negative phenotype. By using their methods, we disclosed that the p53-extreme negative/positive expression was an independent prognostic factor in patients with SDC.

Several immunohistochemical classification systems have been developed as surrogate methods for the molecular subtypes based on the gene expression patterns of breast cancer, which have proved useful in guiding decision-making for systemic therapies, predicting the biological behavior of the tumor, and determining the prognosis [18, 19]. Breast cancer has been proposed to be classified into several subtypes based on the expression profiles of ERα, PR, HER2, and Ki-67 LI, such as luminal A, luminal B, luminal B HER2, HER2-enriched, and triple negative (basal-like) [18, 19]. Unlike breast cancer, however, in SDCs, the expression of ERα and PR is almost exclusively negative [10, 11, 14], while AR is known to be frequently expressed, as noted in our results. Consequently, it is reasonable to postulate that AR expression in SDC is analogous to ERα reactivity in breast cancer, representing an apocrine phenotype. Given the morphologic similarity to mammary ductal carcinoma, two classification systems of SDC based on the biomarker immunoexpression profile were recently proposed [14, 15, 20]. Di Palma et al. suggested that SDC can be classified into four subtypes by a combination of the expression of AR, HER2, EGFR, and CK5/6 as follows: ‘luminal AR positive’, ‘HER2 positive’, and ‘basal-like’ in addition to ‘intermediate’ [14, 20]. They did not take into account the Ki-67 LI in their system and failed to detect a correlation between the nuclear grade and subtype, except that ‘basal-like’ subtype SDCs were high-grade, though no prognostic significance was provided. In the breast cancer classification, the threshold for Ki-67 LI between low- and high-Ki-67 LI groups as 20% is currently accepted by most of experts [36], whereas no optimal cutoff point in SDC has been validated yet. The mean Ki-67 LI of 44% in SDCs was about two times higher than that of breast cancers reported in the literature [37]. Furthermore, survival analysis revealed that the Ki-67 LI value of 40% was the most suitable cutoff point in terms of prognostic relevance. For these reasons, we adopted that value for our SDC classification. In the present study, we refined our previously reported classification system in order to reflect the increased feasibility of an appropriate personalized systemic therapy with anti-HER2, anti-AR, and/or cytotoxic drugs [15]. Consequently, our revised classification system has great advantages in predicting the prognosis of patients with SDC. Di Palma classification is certainly simple for practical use but was only remotely related to the survival by our present case analysis. Since the therapeutic efficacy of our revised classification has yet to be evaluated, further studies are essential to determine its usefulness for devising a relevant treatment strategy for SDC in a clinical setting.

In conclusion, activation of HER family members is more frequently observed in SDC arising in PA than in its *de novo* occurrence. The immunohistochemical expression of AR, CK5/6, and p53 (of its extreme evaluation) was independent prognostic factors in SDC. However, further clinical trials are necessary to establish the optimum treatment referring to the expression profile of SDC. Our classification based on the biomarker immunoprofile was valuable for predicting the survival and might be useful in the future for selecting appropriate therapy for patients with SDC.

## MATERIALS AND METHODS

### Patient selection

This study comprised 151 patients with SDC diagnosed and treated at 7 institutions between 1992 and 2014, except for patients who underwent anti-HER2 or anti-AR therapy as an initial treatment. Those patients include the cases previously reported by Otsuka et al [4]. All tumors were confirmed to have been diagnosed correctly on a central review system by two expert pathologists (T.N. and Y.S.) according to the rigorous histomorphologic criteria for SDC (Figure 1) [1]. Other entities, including high-grade transformation of various carcinomas, high-grade mucoepidermoid carcinoma, squamous cell carcinoma, and adenocarcinoma, not otherwise specified, were carefully eliminated from this study via ancillary analyses, if necessary [7, 28]. Moreover, we conducted histologic review of the multi-step sections from the entire tumor in every case in order to enhance accuracy of identification whether histologic origin of the tumor was *de novo* or ex PA. In the case of SDC ex PA, pre-existing PA component frequently represented a hyalinized nodule surrounded by carcinoma. Even in such an instance, carcinoma nests enclosed within the hyalinized nodule were often rimmed by myoepithelial marker-positive benign neoplastic cells. The patients’ charts were retrospectively appraised to obtain data on the age, gender, tumor site, tumor size, lymph node involvement, distant metastasis, treatment, and outcome. The tumor stage was classified according to the UICC TNM classification and staging system (2010, 7th edition). Most patients were treated surgically with postoperative irradiation and/or chemotherapy.

The present study was approved by the Institutional Ethics Review Board of the ethics committee of each of the seven institutions that participated in this study, and the need to obtain informed consent was waived owing to the retrospective nature of the analysis.

### Immunohistochemistry and FISH

For immunohistochemistry, formalin-fixed, paraffin-embedded tumor tissue was cut into 3-μm-thick sections. A polymer-based detection system with heat-mediated antigen retrieval was conducted using the primary antibodies shown in Supplementary Table 2. Diaminobenzidine was applied to detect antigen-antibody reactions. Appropriate positive and negative controls were employed for all conditions.

Since it has been mentioned that SDC with no immunoreactivity for AR is rare, and that such cases should be carefully diagnosed as SDC [28, 30], in order to ensure the reliability we attempted AR immunohistochemistry repeatedly on multi-step sections for cases when the first trial completely failed immunoreactivity.

To examine the presence of *HER2* amplification, a FISH analysis was carried out for all 151 SDC cases. A 4-μm-thick paraffin section from each block was placed onto a glass slide and subjected to FISH. *HER2* amplification was performed in accordance with the manufacturer's instructions using FISH *HER2* PharmDx (Dako, Glostrup, Denmark), which contained both fluorescently-labeled *HER2* gene and chromosome enumeration probe 17 (CEP17).

### Evaluation of HER2 status

HER2 positivity was defined as either immunohistochemically 3+ or *HER2* amplification according to the American Society of Clinical Oncology/College of American Pathologists (ASCO/CAP) guidelines for breast cancer [27]. Immunohistochemically, HER2 3+ was defined as circumferential membrane staining that was complete, intense, and >10% of tumor cells (Figure 2A). Regarding the FISH analysis, 100 non-overlapping, intact interphase tumor nuclei identified by DAPI staining were evaluated, and the *HER2* gene (red signal) and CEP17 (green signal) copy numbers in each nucleus were assessed. Samples were considered to be amplified when the average copy number ratio (*HER2*/CEP17) was ≥ 2.0 in all nuclei evaluated, or when the *HER2* signals formed a tight gene cluster (Figure 2B).

### Assessment of immunohistochemistry

A case was considered to be positive for AR when ≥ 20% of tumor cell nuclei showed strong staining (Figure 3). The cases were regarded as positive for ERβ when intensely positive staining of the cell nuclei was seen in ≥ 1% of tumor cells [38].

The percentage of EGFR, HER3, MUC1, PLAG1, and CK5/6 immunostaining cells was scored from 0 to 3+ as follows: 0, 0%; 1+, 1% to 10%; 2+, 11% to 30%; and 3+, > 30%. We considered each marker to be positive based on the score level as follows: score 3+ for EGFR (Figure 5A) and MUC1 (Figure 5B); score 1–3+ for HER3 (Figure 5C) and PLAG1; and score 2–3+ for CK5/6 (Figure 5D).

p53 staining results was interpreted based on the expression pattern; cases were classified into three groups in accordance with the methods described in a breast cancer study by Boyle et al. as follows: extreme negative, complete confluent negativity of staining (Figure 6A); extreme positive, strong diffuse confluent positivity (Figure 6B); and non-extreme, all intermediate expression of any intensity [35].

The percentage of Ki-67-positive cells was determined by counting at least 1000 tumor cells, and then recorded as the Ki-67 LI. Ki-67 LI, a value of < 40% was considered to indicate Ki-67-low, while ≥ 40% was considered to indicate Ki-67-high (Figure 4).

### Revised classification based on biomarker immunoprofiling

Referring to the breast cancer immunohistochemical classification as a surrogate for molecular subtyping [18, 19], all SDCs were categorized into four main subtypes based on a combination of the expression of AR (instead of ER or PR for breast cancer), HER2 (or *HER2* amplification status), and Ki-67 as follows: ‘apocrine A’ (AR+/HER2−/Ki-67-low), ‘apocrine B’ (AR+/HER2−/Ki-67-high), ‘apocrine HER2’ (AR+/HER2+), ‘HER2-enriched’ (AR−/HER2+), and ‘double negative’ (AR−/HER2−). The ‘double negative’ subtype was further subclassified into ‘basal-like’ (AR−/HER2−/EGFR and/or CK5/6+) and ‘unclassified’ (others).

### Statistical analyses

The associations between variables in terms of the immunoreactivity were analyzed using a chi-squared test. The OS and PFS rates were evaluated by the Kaplan-Meier method and by univariate and multivariate Cox proportional hazard models adjusted for the age, gender, primary tumor site, TNM classification, first-line treatment, and the histologic origin (*i.e*., *de novo* or ex PA). The association was evaluated based on the hazard ratio and 95% confidence interval. All statistical analyses were performed using the software program STATA ver. 13 (StataCorp, College Station, TX, USA). All tests were two-sided, and *P* values < 0.05 were considered to be statistically significant.

## SUPPLEMENTARY MATERIALS AND TABLES




