# Supplementary material for: Biomarker immunoprofile in salivary duct carcinomas: clinicopathological and prognostic implications with evaluation of the revised classification
Source: Oncotarget. 2017 Aug 2;8(35):59023–35. doi: 10.18632/oncotarget.19812 (PMC5601711; doi:10.18632/oncotarget.19812)
Supplement: Supplementary file 1 [file oncotarget-08-59023-s001.pdf]

Biomarker immunoprofile in salivary duct carcinomas: clinicopathological and prognostic implications with evaluation of the revised classification

SUPPLEMENTARY MATERIALS

Supplementary Table 1: Correlation of biomarker immunoprofile with clinicopathological factors in patients with salivary duct carcinoma. See Supplementary_Table_1

Supplementry Table 2: Antibodies used in this study

Antigen	Clone	Dilution	Source
AR	AR441	R-U	BIOCARE Medical LLC, CA
ERβ	EMR02	x 50	Leica Biosystems, Nusslock, DEU
EGFR	31G7	R-U	NICHIREI Biosciences Inc., Tokyo, JPN
HER2	Polyclonal	x 400	Dako Cytomation, CA
HER3	D22C5	x 50	Cell Signaling Technology, MA
MUC1	Ma695	x 100	Leica Biosystems, Nusslock, DEU
PLAG1	3B7	x 100	Sigma-Aldrich Inc., MO
p53	DO-7	x 200	Dako Cytomation, CA
CK5/6	D5/16B4	x 50	Dako Cytomation, CA
Ki-67	MIB-1	x 100	Dako Cytomation, CA

Abbreviations: AR = androgen receptor; ER = estrogen receptor; EGFR = epidermal growth factor receptor; HER = human epidermal growth factor receptor; MUC1 = mucin-1; PLAG1 = pleomorphic adenoma gene 1; CK = cytokeratin; and R-U = ready-to-use.
