# Supplementary material for: Biomarker immunoprofile in salivary duct carcinomas: clinicopathological and prognostic implications with evaluation of the revised classification
Source: Oncotarget. 2017 Aug 2;8(35):59023–35. doi: 10.18632/oncotarget.19812 (PMC5601711; doi:10.18632/oncotarget.19812)
Supplement: Supplementary file 2 [file oncotarget-08-59023-s002.docx]

| **Supplementary Table 1:** Correlation of biomarker immunoprofile with clinicopathological factors in patients with salivary duct carcinoma | | | | | | | | | | | | | | | | | | | | | | | | | | |  |  |  |  |  |  |  |  |  |  |  |  |  |
| --- | --- | --- | --- | --- | --- | --- | --- | --- | --- | --- | --- | --- | --- | --- | --- | --- | --- | --- | --- | --- | --- | --- | --- | --- | --- | --- | --- | --- | --- | --- | --- | --- | --- | --- | --- | --- | --- | --- | --- |
|  | **AR** | | |  | **ERβ** | | |  | **EGFR** | | |  | **HER2** | | |  | **HER3** | | |  | **MUC1** | | |  | **PLAG1** | | |  | **p53** | | |  | **CK5/6** | | |  | **Ki-67** | | |
|  | **Neg** | **Pos** | ***P*** |  | **Neg** | **Pos** | ***P*** |  | **Neg** | **Pos** | ***P*** |  | **Neg** | **Pos** | ***P*** |  | **Neg** | **Pos** | ***P*** |  | **Neg** | **Pos** | ***P*** |  | **Neg** | **Pos** | ***P*** |  | **NE** | **EN/EP** | ***P*** |  | **Neg** | **Pos** | ***P*** |  | **Low** | **High** | ***P*** |
| Age, y |  |  | N.S. |  |  |  | N.S. |  |  |  | N.S. |  |  |  | N.S. |  |  |  | N.S. |  |  |  | N.S. |  |  |  | N.S. |  |  |  | N.S. |  |  |  | N.S. |  |  |  | N.S. |
| <65 | 21 | 63 |  |  | 3 | 78 |  |  | 57 | 27 |  |  | 43 | 41 |  |  | 28 | 56 |  |  | 20 | 61 |  |  | 39 | 42 |  |  | 46 | 36 |  |  | 57 | 25 |  |  | 32 | 52 |  |
| ≥65 | 12 | 54 |  |  | 2 | 65 |  |  | 44 | 23 |  |  | 38 | 29 |  |  | 20 | 46 |  |  | 13 | 54 |  |  | 29 | 38 |  |  | 38 | 29 |  |  | 47 | 20 |  |  | 32 | 35 |  |
| Gender |  |  | 0.011* |  |  |  | N.S. |  |  |  | N.S. |  |  |  | 0.002* |  |  |  | N.S. |  |  |  | N.S. |  |  |  | N.S. |  |  |  | N.S. |  |  |  | N.S. |  |  |  | N.S. |
| Male | 23 | 103 |  |  | 4 | 120 |  |  | 81 | 46 |  |  | 75 | 52 |  |  | 40 | 86 |  |  | 28 | 96 |  |  | 57 | 67 |  |  | 72 | 53 |  |  | 86 | 39 |  |  | 53 | 74 |  |
| Female | 10 | 14 |  |  | 1 | 23 |  |  | 20 | 4 |  |  | 6 | 18 |  |  | 8 | 16 |  |  | 5 | 19 |  |  | 11 | 13 |  |  | 12 | 12 |  |  | 18 | 6 |  |  | 11 | 13 |  |
| T classification |  |  | 0.021* |  |  |  | N.S. |  |  |  | N.S. |  |  |  | N.S. |  |  |  | 0.038* |  |  |  | N.S. |  |  |  | N.S. |  |  |  | N.S. |  |  |  | N.S. |  |  |  | N.S. |
| 1-3 | 12 | 69 |  |  | 2 | 78 |  |  | 55 | 27 |  |  | 40 | 42 |  |  | 20 | 61 |  |  | 14 | 66 |  |  | 38 | 42 |  |  | 48 | 33 |  |  | 56 | 24 |  |  | 35 | 47 |  |
| 4 | 21 | 48 |  |  | 3 | 65 |  |  | 46 | 23 |  |  | 41 | 28 |  |  | 28 | 41 |  |  | 19 | 49 |  |  | 30 | 38 |  |  | 36 | 32 |  |  | 48 | 21 |  |  | 29 | 40 |  |
| N classification |  |  | N.S. |  |  |  | N.S. |  |  |  | N.S. |  |  |  | N.S. |  |  |  | N.S. |  |  |  | N.S. |  |  |  | N.S. |  |  |  | 0.005* |  |  |  | N.S. |  |  |  | 0.023* |
| 0 | 11 | 59 |  |  | 3 | 66 |  |  | 49 | 22 |  |  | 35 | 36 |  |  | 21 | 50 |  |  | 14 | 55 |  |  | 31 | 38 |  |  | 48 | 22 |  |  | 53 | 16 |  |  | 37 | 34 |  |
| 1-3 | 22 | 58 |  |  | 2 | 77 |  |  | 52 | 28 |  |  | 46 | 34 |  |  | 27 | 52 |  |  | 19 | 60 |  |  | 37 | 42 |  |  | 36 | 43 |  |  | 51 | 29 |  |  | 27 | 53 |  |
| M classification |  |  | N.S. |  |  |  | N.S. |  |  |  | N.S. |  |  |  | N.S. |  |  |  | N.S. |  |  |  | N.S. |  |  |  | N.S. |  |  |  | N.S. |  |  |  | N.S. |  |  |  | 0.008* |
| 0 | 31 | 110 |  |  | 5 | 134 |  |  | 96 | 46 |  |  | 78 | 64 |  |  | 46 | 95 |  |  | 30 | 109 |  |  | 62 | 77 |  |  | 80 | 60 |  |  | 98 | 42 |  |  | 64 | 78 |  |
| 1 | 2 | 7 |  |  | 0 | 9 |  |  | 5 | 4 |  |  | 3 | 6 |  |  | 2 | 7 |  |  | 3 | 6 |  |  | 6 | 3 |  |  | 4 | 5 |  |  | 6 | 3 |  |  | 0 | 9 |  |
| Primary site |  |  | 0.029* |  |  |  | N.S. |  |  |  | 0.030* |  |  |  | N.S. |  |  |  | N.S. |  |  |  | N.S. |  |  |  | N.S. |  |  |  | N.S. |  |  |  | N.S. |  |  |  | N.S. |
| Parotid gland | 30 | 87 |  |  | 3 | 112 |  |  | 83 | 34 |  |  | 64 | 53 |  |  | 41 | 75 |  |  | 25 | 90 |  |  | 52 | 63 |  |  | 69 | 47 |  |  | 80 | 36 |  |  | 52 | 65 |  |
| Submandibular gland | 2 | 27 |  |  | 1 | 28 |  |  | 15 | 15 |  |  | 16 | 14 |  |  | 6 | 24 |  |  | 7 | 22 |  |  | 15 | 14 |  |  | 13 | 16 |  |  | 20 | 9 |  |  | 10 | 20 |  |
| Histologic origin |  |  | N.S. |  |  |  | N.S. |  |  |  | 0.015* |  |  |  | <0.001* |  |  |  | 0.045* |  |  |  | N.S. |  |  |  | N.S. |  |  |  | N.S. |  |  |  | N.S. |  |  |  | 0.020* |
| *De novo* | 12 | 45 |  |  | 3 | 52 |  |  | 45 | 12 |  |  | 43 | 14 |  |  | 24 | 33 |  |  | 14 | 41 |  |  | 25 | 30 |  |  | 37 | 19 |  |  | 42 | 14 |  |  | 31 | 26 |  |
| Ex pleomorphic adenoma | 20 | 68 |  |  | 2 | 86 |  |  | 53 | 36 |  |  | 35 | 54 |  |  | 23 | 65 |  |  | 18 | 70 |  |  | 41 | 47 |  |  | 44 | 44 |  |  | 59 | 29 |  |  | 31 | 58 |  |
| Abbreviations: AR = androgen receptor; ER = estrogen receptor; EGFR = epidermal growth factor receptor; HER = human epidermal growth factor receptor; MUC1 = mucin-1; PLAG1 = pleomorphic adenoma gene 1; CK = cytokeratin; Neg = negative; Pos = positive; NE = not extreme; EN/EP = extreme negative/positive; N.S. = not significant. | | | | | | | | | | | | | | | | | | | | | | | | | | | | | | | | | | | | | | | |
| *Statistically significant (*P*<0.05) | |  |  |  |  |  |  |  |  |  |  |  |  |  |  |  |  |  |  |  |  |  |  |  |  |  |  |  |  |  |  |  |  |  |  |  |  |  |  |
